# Testicular Adrenal Rest Tumor in Two Brothers with a Novel Mutation in the 3-Beta-Hydroxysteroid Dehydrogenase-2 Gene

**DOI:** 10.4274/jcrpe.3306

**Published:** 2017-03-01

**Authors:** Ayla Güven, Seher Polat

**Affiliations:** 1 Göztepe Training and Research Hospital, Clinic of Pediatric Endocrinology, İstanbul, Turkey; 2 Amasya University Faculty of Medicine, Department of Pediatrics, Amasya, Turkey; 3 Erciyes University Faculty of Medicine, Department of Medical Genetics, Kayseri, Turkey

**Keywords:** HSD3β gene, testicular adrenal rest tumor, congenital adrenal hyperplasia, 46, XY disorder of sex development

## Abstract

Testicular adrenal rest tumors (TART) occur frequently in adolescents and adults with 21-hydroxylase deficiency. There have been no reports of TART in children with 3β-hydroxysteroid dehydrogenase deficiency (HSD3β). Biopsy proven TART was diagnosed in a 3^1/12^-year-old male patient and also in his 22-month-old sibling. Hormonal and anthropometric measurements were performed during glucocorticoid and fludrocortisone treatment. The mutational analysis was performed by direct DNA sequencing of the complete coding region of the *HSD3β2* gene. Initially, both siblings were treated with high doses of hydrocortisone and fludrocortisone. TART regressed with dexamethasone treatment in both patients. However, growth velocity decreased and weight gain increased in both patients. Dexamethasone was changed to high-dose hydrocortisone (>20 mg/m^2^/d). Sequencing analyses revealed a novel homozygous p.W355R (c.763 T>C) mutation at exon 4 of the *HSD3β2* gene in both siblings. These two patients are, to our knowledge, the first known cases of TARTs with a novel mutation in the *HSD3β2* gene detected during childhood. High-dose hydrocortisone treatment is more reliable for TART in children.

**WHAT IS ALREADY KNOWN ON THIS TOPIC?**

Testicular adrenal rest tumors (TART) are usually seen in adolescents and adults with congenital adrenal hyperplasia (CAH) due to 21-hydroxlase deficiency.

**WHAT THIS STUDY ADDS?**

We presented two siblings with CAH due to novel mutation in 3β-hydroxysteroid dehydrogenase gene. To the best of our knowledge, TART development in children and infant with novel 3v-Beta-Hydroxysteroid Dehydrogenase-2 mutation has not been reported previously.

## INTRODUCTION

Testicular adrenal rest tumors (TART) are frequently encountered in adult male patients with congenital adrenal hyperplasia (CAH) caused by 21-hydroxylase deficiency (21-OHD). TART can also be detected in early childhood. The youngest CAH patient with TART reported in the literature was younger than 8 weeks old (1,2).

To the best of our knowledge, the two patients presented in this report are the youngest cases of 3-beta-hydroxysteroid dehydrogenase (HSD3β) deficiency associated with TART.

## CASE REPORTS

### Case 1

A 17-day-old baby boy was referred to our Department from another clinic with hypospadias, left cryptorchidism, and bifid scrotum. His parents were second cousins. His birth weight was 3600 g and his length was 52 cm. Karyotype was 46,XY. Laboratory investigation was consistent with adrenal insufficiency: sodium (Na) 120 mEq/L, potassium (K) 6.9 mEq/L, chlorine (Cl) 96 mEq/L, adrenocorticotropic hormone (ACTH) 546 pg/mL, 17-hydroxyprogesterone (17-OHP) 29 ng/mL, and dehydroepiandrosterone sulfate (DHEAS) 1550 μg/dL. In addition to fluids and electrolytes, hydrocortisone (HC) (100 mg/m^2^/d, divided into three doses, i.v.) and fludrocortisone (FC) (0.1 mgx2/d, p.o.) were added to the treatment. At age 6 months, HC and FC treatment was stopped for a week after which an ACTH-stimulation test was performed. The test results were consistent with HSD3β (Table 1). The parents were told to bring the patient for a follow-up visit every 3 months. During the follow-up period, the daily dose of HC was continued as higher than 13 mg/m^2^ (p.o., divided into three doses, with the morning dose being highest) and the serum ACTH level was below 42 pg/mL. However, plasma renin activity (PRA) was higher for his age at 19 months old (77.6 ng/mL/hr, normal: 1.71-11.15). Despite strict treatment with FC, increased PRA levels for his age were found to persist.

**Figure 1:**
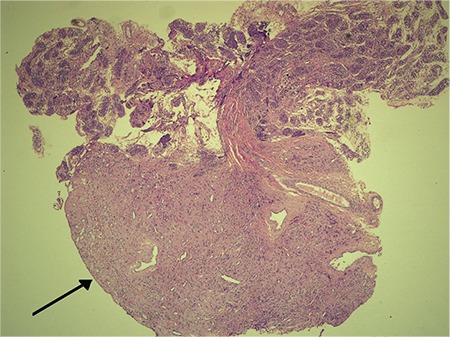
Leydig cell proliferation area (marked with a black arrow) and adjacent residual testicular parenchyma predominantly composed of Sertoli cells (HE x20)

**Figure 2:**
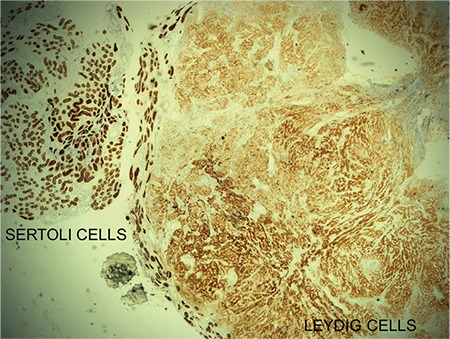
Leydig and Sertoli cells showed immunopositivity with inhibin B

The patient underwent a hypospadias and left orchidopexy operation at age 15 months. At 31/12 years of age, the right testicle volume was 4 mL. ACTH level was 42 pg/mL and PRA 29.1 pg/mL (1-6.5). Scrotal ultrasonography (USG) showed that the diameters for the right testicle were 33x15x13 mm (3.5 mL) and 11x11x6 mm (0.4 mL) for the left testicle. Heterogeneous parenchyma and multiple different-sized hypoechoic nodules associated with microcalcifications were noted in the right testicle. Although the increased right testicular volume was thought to be due to TART, the pediatric surgeon performed a biopsy to rule out a Leydig cell tumor. Pathologic examination of the right testicle revealed diffuse Leydig cell proliferation (Figure 1). Reinke crystalloids were not identified. Strong immunopositivity for inhibin B was detected in the tissue (Figure 2). TART was diagnosed, and HC was changed to dexamethasone (DEX) (0.5 mg/day, p.o. administered in a single late-evening dose). DEX was used to optimize medical treatment in a dosage equivalent to HC [20 mg of HC=0.5 mg DEX (3)]. After 6 months of suppressive DEX treatment, growth velocity (GV) was 0.5 cm and the patient became obese [body mass index standard deviation score (BMI SDS) 2.77]. Therefore, DEX was changed to prednisolone (6 mg/m^2^/day). Although GV of the patient improved with prednisolone treatment, the nodules failed to regress. During prednisolone treatment, ACTH and PRA were within normal ranges (19.2 pg/mL and 2.1 ng/mL/h, respectively). When he was 5 years old, although plasma ACTH was normal (8.8 pg/mL), PRA was increased (12.3 ng/mL/h, normal: 0.5-5.85) and high-dose HC (20 mg/m^2^/day) was started again. To check the efficiency of the treatment, anthropometric measurements and physical examinations were performed and ACTH, 1.4 androstenedione, and testosterone levels were measured at three-month intervals. During the treatment, GV was calculated as 7.5 cm, 7.2 cm, and 5 cm for the first, second, and third years, respectively.

At 8 years of age, hypoechoic masses with irregular contour in both testicles were found in scrotal USG examination (34x14x9 mm in the right testicle and 13x8x4 mm in the left testicle). High-dose HC was switched to DEX. DEX and FC treatment was continued until the patient was 810/12-year-old, at which time, the DEX treatment was stopped and high-dose HC treatment was started again. At the last visit, he was 9^4/12^-year-old. Weight was 38.4 kg (1.22 SD), height was 137.2 cm (0.27 SD), BMI was 20.1 kg/m^2^ (1.38 SD), and bone age was 10 years. The right testicle was 5 mL, left testicle was 2 mL. GV was 6.2 cm. A scrotal USG revealed regression of TART in the right testicle (hypoechoic mass 4x4x5 mm). The results of hormonal analyses at this time were: ACTH 49 pg/mL, androstenedione <0.3 ng/mL, and PRA 3.1 ng/mL/h.

### Case 2

This patient was the younger brother of the first patient. The patient was admitted to clinic for hypospadias at age 4 days. His birth weight was 3300 g and length was 51 cm. At admission, weight was 3000 g. The phallus was 2x1.5 cm on the dorsal side and 1.5x1.5 cm on the ventral side. Urethral meatus was opening to the base of the phallus. Bilateral testicles were detected in the bifid scrotum. The results of the ACTH-stimulation test performed at age 10 days revealed HSD3β deficiency (Table 1). The karyotype of the patient was 46,XY. HC and FC were started. His physical and laboratory examinations were repeated every 3 months. At age 22 months, both testes volumes were found to be increased for age (left 3 mL, right 5 mL). This finding was accompanied by a high plasma ACTH level (161 pg/mL, normal: 6-46) and a normal PRC level (16.8 ng/mL, normal: 1.71-11.15). Scrotal USG showed that the right testicle was 28x11x10 mm and the left one was 28x12x9 mm. Also, hypoechoic masses, 21x8x6 mm in diameter, were detected in the hilum in both testicles. TART was diagnosed, and the HC dose was increased to 20 mg/m^2^/day. After 4 months, testicular size was reduced to 2 mL. The HC dose was decreased to 15 mg/m^2^/day. The patient underwent a hypospadias operation at age 26/12 years. At age 211/12 years, both testicles were increased in size to 6 mL. HC was substituted with DEX in a dose of 0.5 mg/day (=30 mg/d HC). High-dose DEX treatment was given for three months, following which, the dose was decreased to 0.35 mg/d. DEX and FC treatment was maintained for 11 months, then, because the patient was becoming obese (BMI SD 3.87), the treatment was switched to high-dose HC (25 mg/m^2^/day). During DEX treatment, GV was 5.4 cm/year. Bone age was 4 years. The efficiency of the treatment was also checked by measuring ACTH, 1.4 androstenedione, and testosterone levels at three-month intervals. On his last visit, the patient was 4^8/12^-year-old, his weight was 27 kg (2.73 SD), his height was 110.1 cm (0.69 SD), the volume of the right testicle was 3 mL, and that of the left testicle was 1 mL. During high-dose HC treatment, GV was 7.1 cm/ 9 months. Scrotal USG revealed a 2x2x1-mm hypoechoic mass in the right testicle. Hormonal analyses revealed an ACTH level of 7.3 pg/mL (< 46), serum 1.4 androstenedione level of <0.3 ng/mL, and PRA was 8.06 ng/mL/h (0.5-5.85).

### Hormonal Analysis

Morning blood samples were obtained from both siblings for basal androgens and precursor levels. ACTH-stimulation test (Synacten, 250 μg intravenously) was also performed. Total testosterone, DHEAS, cortisol, and ACTH were estimated using immunoenzymatic methods (Beckman Coulter, DXI 800 ,USA). 17-OH progesterone and 1,4 androstenedione, aldosterone, and PRA were measured using an Immunotech assay kit (Beckman Coulter) with radioimmunoassay method (ICN ISO DATA Gamma Counter).

### Imaging Studies

The same investigator performed TART investigation by using ultrasonography and the Toshiba aplio XV 3.5 MHz convex probe. Measurements were performed separately for the right and left testicles. Testicular volume was calculated with the formula [(length X width (mm) × height (mm) × 0.523/1000 (mL)].

### Mutational Analysis of *HSD3β2*

Blood samples for DNA analysis were obtained after having an informed consent from the parents. A standard protocol was followed for the preparation of genomic DNA from peripheral blood leukocytes. Exons II, III, and IV, and the exon-intron boundaries of the *HSD3β2* gene were amplified by polymerase chain reaction as described previously (4). The mutational analysis was performed by direct DNA sequencing of the complete coding region of the *HSD3β2* gene. The samples were electrophoresed on an automated sequencer (ABI3500) and analysed with the ABI SeqScape 3.7 software. Sequence variants were designated according to the recommendations of the Human Genome Variation Society (www.hgvs.org/rec.html) using the GenBank reference sequences. NC_000001.11 (HSD3β2 g.DNA), NM_000198.3 and (HSD3β2 c.DNA), NP_000189.1 (HSD3β2 p.protein).

**Figure 3:**
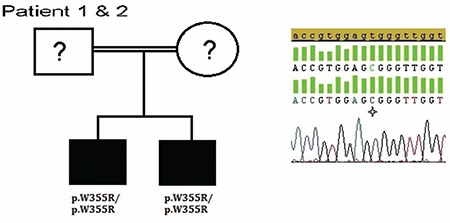
Molecular genetic analysis of the HSD3β2 gene. Pedigree of the patients with electropherograms of the mutation [p.W355R (c.763 T>C)]. Star indicates mutated nucleotides. Question marks indicate individuals not available for genetic analysis

Sequencing analyses revealed a novel homozygous p.W355R (c.763T>C) mutation (Figure 3) located at exon 4 of the HSD3β2 gene in both siblings. Parents’ DNA samples were not available for genetic studies.

## DISCUSSION

This report of two patients describes TART development in two brothers with an HSD3β2 homozygous mutation. Bilateral TART associated with large adrenal rest tumor located in the perirenal region was reported in an adult CAH patient with known HSD3β2 mutation (5). However, TART development in infants/children has not been reported previously.

TART is most frequently seen in adult males with CAH. Presence of adrenal gland masses in the testis in childhood was first demonstrated in 1940 in a 37/12-year-old child with CAH (6). The youngest patient reported in the literature was a 3-week-old infant who died due to Adrenogenital syndrome (7).

In almost all cases with TART, the tumor is associated with 21-OHD (1). Frequency of TART gradually increases during the pubertal period, and its prevalence in 21-OHD was reported as 28% in early puberty and as 100% at the end of puberty (8). On the other hand, TART is rarely reported in 11-hydroxylase-deficient patients (9). To the best of our knowledge, these two siblings presented here are the youngest cases of HSD3β2 mutation associated with TART.

The etiology and pathogenesis of TART in CAH patients are not completely understood. In the embryonic period, ectopic adrenocortical cells are neighbors of the testes. These cells, along with the testicles, can also be stimulated by ACTH during the descent of the testicles and are thought to have migrated into the scrotum during this early period (10).

It has been previously accepted that ACTH has the most important role in TART growth. Because these tumors usually develop in poorly-controlled patients, the increased ACTH is thought to stimulate the proliferation of these tumors. In fact, intensive glucocorticoid treatment may reduce the tumor size in most cases (10,11). However, occurrence of TART in well-controlled CAH patients and the observations that glucocorticoids in supra-physiologic doses cannot reduce these tumors in some patients are findings which indicate that other growth-promoting factors possibly play a role in the pathogenesis of TART (12).

It has been shown that CYP11B1 and CYP11B2’s mRNAs involved in the synthesis of aldosterone are excessively expressed in TART (13,14). Also ACTH and mRNA expression of angiotensin II (AII) receptors in the adrenal rest tissues was detected (15). AII has a strong trophic effect on the zona glomerulosa and stimulates aldosterone synthesis (16). If efficient suppression of renin is not done, angiotensinogen I production could be stimulated and, in salt-wasting CAH patients, AII synthesis could be increased. Consequently, ectopic steroidogenic cells in the testes would increase and could lead to TART.

Leydig cell tumor shows a similar histopathologic appearance with specific features of TART. Testicular tumor of CAH is frequently bilateral, but Leydig cell tumor is commonly unilateral.

Long-term and high-dose glucocorticoids (such as DEX) cause severe adverse effects such as obesity, osteoporosis, disorders in glucose metabolism, and growth retardation in children. On the other hand, high-dose glucocorticoids may suppress ACTH level and can cause TART regression (1,10). Single-dose DEX with or without additional HC doses associated with FC constitute the current treatment options for TART. Although some patients do not accept this treatment, still we need to use DEX to regress TART in some patients (10,11). On the other hand, these lesions may increase in size and number when glucocorticoid dose is decreased. Mineralocorticoid therapy is often underestimated in the management of TART, but the suppression of renin by adequate FC treatment, and thus contributing to reduced production of AII, is important in preventing TART development.

In both siblings, firstly, the HC dose was increased to more than 20 mg/m^2^/d. However, the TART tissue did not show the expected regression, so HC was switched to DEX. Significant regression in TART tissue was obtained. During treatment with DEX, mild bone age advancement was noted in both patients. Growth rate decreased in both and they became obese. Therefore, DEX treatment was switched back to HC. Fortunately, normal GV was achieved with HC treatment in both siblings.

Although some authors recommend USG screening in all males with CAH from the age of 8, the age of TART screening in childhood is still unclear (17). We believe that regardless of age, all boys with CAH should undergo a careful physical examination, and when TART is suspected, scrotal USG examination should be performed. TART needs to be recognized at an early stage to prevent the adverse effects of aggressive glucocorticoid therapy.

The novel p.W355R mutation located in the C-terminal part of the protein and in vitro expression studies related with C-terminal part of the protein showed that the two truncated p.R335X, p.W355X mutant proteins yielded absent conversion of pregnenolone and dehydroepiandrosterone (DHEA), whereas the missense mutation p.P341L showed a residual DHEA conversion of 6% of wild-type activity. It was thus concluded that C-terminal mutations of the HSD3β2 gene are responsible for classical *HSD3β2* deficiency due to putative structural alteration of the HSD3β2 protein and that this process is further aggravated by increased protein degradation (18). In our cases, two siblings with p.W355R mutation showed classical *HSD3β2* deficiency; therefore our study also shows that the C-terminal part of the protein must be important for correct enzymatic function.

Our observations in these two patients suggest that TART may develop at any age in CAH patients. In addition, these brothers are also interesting in that they are the youngest cases with novel HSD3β2 mutation reported in the literature.

## Figures and Tables

**Table 1:**
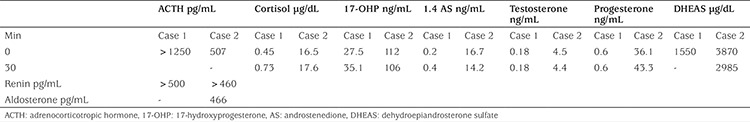
Adrenocorticotropic hormone-stimulation test results in the two patients (blood levels)
